# A Patient With Miller Fisher Syndrome With Positive GQ1b and Aquaporin-4 Antibodies: Will There Be an Aquaporin-4 Antibody Associated Disorder?

**DOI:** 10.7759/cureus.43428

**Published:** 2023-08-13

**Authors:** Fatin Aylia, Karn Johri, Riley Spencer, David Chu, Mehron Deriss, Davin Peng, Jonathan Eskenazi, Antonio K Liu

**Affiliations:** 1 Internal Medicine, Adventist Health White Memorial, Los Angeles, USA; 2 Family Medicine, Charleston Area Medical Center, Charleston, USA; 3 Neurology, Ross University School of Medicine, Miramar, USA; 4 Neurology, Adventist Health White Memorial, Los Angeles, USA; 5 Neurology, California Hospital Medical Center, Los Angeles, USA

**Keywords:** myelin-oligodendrocyte glycoprotein (mog), aquaporin 4, gq1b, neuromyelitis optica spectrum disorder, miller fisher syndrome (mfs)

## Abstract

There have been many advancements in the field of neuromyelitis optica and neuromyelitis optica spectrum disorder since the discovery of aquaporin-4 (AQP4) and myelin oligodendrocyte glycoprotein antibodies. It is also recognized that the pathological features associated with myelin oligodendrocyte glycoprotein antibodies are beyond the domain of neuromyelitis optica spectrum disorder and there is a separate nomenclature, namely myelin oligodendrocyte glycoprotein antibody associated disease. Currently, there is no aquaporin-4 antibody associated disorder, even though aquaporin-4 antibodies are not as widely present in other disorders.

Miller Fisher syndrome (MFS) is a variant of Guillain Barré syndrome, in which there are positive GQ1b antibodies with no evidence of myelitis or optic neuritis. MFS is not considered a component of neuromyelitis optica spectrum disorder. We report on a patient with MFS that was positive for GQ1b and aquaporin-4 antibodies but negative for myelin oligodendrocyte glycoprotein antibodies and is devoid of any features of neuromyelitis optica spectrum disorder. This finding may lead to investigations and reports of other pathologies that are associated with the aquaporin-4 antibody.

## Introduction

Neuromyelitis optica (NMO) is a syndrome characterized by optic neuritis and myelitis covering more than three spinal levels [1]. For a long time, it was recognized as Devic’s disease, which was believed to be a variant of multiple sclerosis (MS). The identification of aquaporin-4 (AQP4) antibody firmly established NMO as a completely different disease entity from MS with its own pathogenesis, treatment, and prognosis [2]. Since then, newer features have been recognized as a component of the disease, hence NMO evolved into neuromyelitis optica spectrum disorder (NMOSD). Newer clinical features include area postrema syndrome, brainstem syndrome, diencephalic syndrome, and cortical lesions [3]. Besides the AQP4 antibody, the myelin oligodendrocyte glycoprotein (MOG) antibody was discovered in many NMO patients that were seronegative for the AQP4 antibody [4]. Since the clinical features and outcome of this group of patients differ enough from the NMOSD group, a new diagnosis emerged and has been labeled the myelin oligodendrocyte glycoprotein antibody associated disorder (MOGAD) [5]. Its criteria and diagnostic requirements are currently being developed. MOGAD differs from NMOSD in that it is mainly found in acute demyelinating encephalomyelitis (ADEM) and in children.

AQP4 is present abundantly in the brain on the astrocytic membrane, NMOSD with seropositive AQP4 is known to be an autoimmune astrocytopathy [6]. MOG antibody, on the other hand, attacks myelin. Pathologic features of perivenous inflammatory demyelination with MOG-dominant myelin loss are key features setting MOGAD apart from MS and AQP4-positive NMOSD [5]. It is important to make the correct diagnosis as the clinical course, treatment and prognosis differ between these disorders. Unlike the MOG antibody, AQP4 is not commonly found in other disease manifestations; there is no known association between AQP4 and Miller-Fisher syndrome (MFS).

MFS is a well-established variant of Guillain Barré syndrome (GBS), featuring descending weakness, ophthalmoplegia, ataxia, and decreased deep tendon reflex [7]. Treatment usually includes immune modulation, and most patients experience improvement. From an immunological standpoint, prior to 2020, GQ1b antibody is common association with one study citing an 85% sensitivity [8]. However, during the current pandemic, many studies have cited seronegative findings with respect to GQ1b [9,10]. This reinforces the idea that commonly tested ganglioside antibodies are only a single cause of this heterogenous group of demyelinating neuropathies.

In our case of a patient with MFS, both GQ1b and AQP4 antibodies were positive. What role does AQP4 play in MFS? Is AQP4 present in other disease entities? 

## Case presentation

A 23-year-old female with no significant past medical history presented to our emergency department with a headache, pain with extraocular movements, and progressive binocular diplopia for five to six days. There was no recent illness preceding her symptoms. She had no COVID-19 infection and received no recent COVID-19 vaccination. On physical examination, she was coherent, pleasant, cooperative, and oriented. She was afebrile and her vital signs were within normal limits. On examination of her cranial nerves, her pupils were equal and reactive to light, and her visual acuity was normal when each eye was examined separately, however, there was bilateral ptosis. The patient was unable to look up or down. On horizontal gaze, the right eye has no movement at all, while the left eye was able to abduct and adduct minimally. There was no facial droop, no dysarthria, and no dysphagia. While she had 5/5 motor strength, she was unable to stand or walk without assistance. She had diminished reflexes and mild dysmetria on finger-to-nose examination. There was marked dysdiadochokinesia. Sensory examination was unremarkable. There was no urine or bowel incontinence. MRI of her brain and her whole spine with and without contrast were all negative. There was no evidence of white matter disease or myelitis. Cerebral spinal fluid (CSF) analysis showed three white blood cells per mm3 and protein of 57 mg/dL (range 14 -40). All other CSF labs, cultures, and studies including a CSF multiple sclerosis panel were negative. Serum vitamin B12, thyroid stimulating hormone, folate, autoimmune neurological diseases reflexive panel, and MOG antibodies were all negative. Serum laboratory testing revealed positive GQ1b antibodies (as the sole positive ganglioside antibodies) at 368 IV (range 0 - 50) and positive AQP4 antibodies of 3.1 U/mL (range 0-2.9).

The patient received a course of 1000 mg intravenous solu-medrol daily for five days and intravenous immunoglobulin (IVIG) 400 mg/kg/day daily for five days. Her symptoms rapidly improved and she was asymptomatic three weeks after presentation.

## Discussion

The diagnosis of MFS was established as the patient had the classic symptoms of ophthalmoplegia, ataxia, and diminished reflexes. GQ1b antibodies were also significantly elevated. The patient’s symptoms had the clinical course, time frame, and therapeutic response that is typical of MFS. There were no features of NMOSD or MOGAD, making a co-existing diagnosis or an overlapping syndrome less likely.

MFS has been most commonly reported with GQ1b antibodies; however, the immunological pattern of MFS has significant variability. More importantly, MFS remains a clinical diagnosis, as GQ1b antibody testing is not sufficiently sensitive, and often no particular antibodies can be identified. In other situations, MFS has been found to be associated with other ganglioside antibodies other than GQ1b, such as GM1, GD1a, GT1a, or combinations of the aforementioned antibodies [11,12]. Besides ganglioside antibodies, multiple studies have reported glutamic acid decarboxylase (GAD) antibodies being present [12,13,14]. During the current COVID-19 pandemic, as well as the vaccination programs, MFS symptoms have been described. However, the detection of GQ1b antibodies in these cases is rare [9,10]. All these observations may suggest the clinical triad of ophthalmoplegia, ataxia, and areflexia as a common final manifestation of a demyelinating pathology. A demyelinating pathology with multiple possible immunological triggers, and some “entry points” that are more common than others.

Upon review of the existing literature, there is no case report of MFS with positive AQP4 antibodies. Nor is there literature endorsing the presence of both AQP4 and GQ1b antibodies in the same disease entity. One case study reported a patient with NMOSD who presented with bilateral INO and AQP4 antibodies, masquerading as MFS. However, GQ1b antibodies were negative [15]. Cases of internuclear ophthalmoplegia with AQP4 antibodies are present in the literature. However, none of them have reported positive GQ1b antibodies. They were all classified as NMOSD [16,17,18]. Since our patient had symptoms only consistent with MFS, her diagnosis would undoubtedly remain MFS in a “pre-antibody testing” era. Classifying it as part of NMOSD would be inappropriate simply based on the presence of AQP4 antibodies.

AQP4 antibody has seldom been reported in other disease entities. When it is reported, it usually overlaps or co-exists with some form of NMOSD; not free of NMOSD features. Among these reported diseases are Sjogren’s syndrome, myasthenia gravis (MG), and N-methyl-D-aspartate receptor encephalitis (NMDAR) [19,20,21]. One report even had three different types of antibodies being positive in an NMOSD, MG, and NMDAR co-existing situation [22]. All reports focused on the “co-existing” aspect of the pathology; no attempts were made to investigate if this could be an AQP4-associated disorder.

## Conclusions

In this case study, we documented the presence of both GQ1b and AQP4 antibodies in a patient with MFS. From a disease standpoint, MFS, GBS, NMOSD, and MOGAD are all clinical diagnoses helped by the presence of their respective antibodies; none of which has a 99% sensitivity. From the individual antibody standpoint, GQ1b, AQP4, and MOG antibodies can all manifest in different manners. MOG has a MOGAD already; AQP4 has fewer reported disease manifestations besides NMOSD. Our case report is an interesting observation of detecting AQP4 antibodies in one patient with MFS; it is far from demonstrating any association. With increasing testing for AQP4 antibodies, more disease entities will likely be found with positive AQP4. Therefore, consideration of the possibility of an AQP4-associated disorder may be warranted.
